# Supplementary material for: Modeling glioblastoma heterogeneity as a dynamic network of cell states
Source: Mol Syst Biol. 2021 Sep 16;17(9):e10105. doi: 10.15252/msb.202010105 (PMC8444284; doi:10.15252/msb.202010105)
Supplement: Supplementary file 6 — Source Data for Figure 5 [file MSB-17-e10105-s004.zip › Figure5A_sourcedata/GSEA_3071/nosigdb_stateIII.GseaPreranked.1622795155562/LARSSON_STATE_2.html]

Details for gene set LARSSON\_STATE\_2[GSEA]

|  || Dataset | state33071 |
| Phenotype | NoPhenotypeAvailable |
| Upregulated in class | na\_neg |
| GeneSet | LARSSON\_STATE\_2 |
| Enrichment Score (ES) | -0.59089124 |
| Normalized Enrichment Score (NES) | -3.1312282 |
| Nominal p-value | 0.0 |
| FDR q-value | 0.0 |
| FWER p-Value | 0.0 |
Table: GSEA Results Summary

  

Fig 1: Enrichment plot: LARSSON\_STATE\_2      
 Profile of the Running ES Score & Positions of GeneSet Members on the Rank Ordered List

  

| PROBE | GENE SYMBOL | GENE\_TITLE | RANK IN GENE LIST | RANK METRIC SCORE | RUNNING ES | CORE ENRICHMENT || 1 | ACSL3 |  |  | 529 | -0.256 | -0.1278 | No |
| 2 | ARGLU1 |  |  | 633 | -0.261 | -0.1456 | No |
| 3 | DDOST |  |  | 1114 | -0.285 | -0.2598 | No |
| 4 | LAPTM4A |  |  | 1391 | -0.299 | -0.3209 | No |
| 5 | XRCC2 |  |  | 1435 | -0.302 | -0.3219 | No |
| 6 | CALR |  |  | 1498 | -0.306 | -0.3276 | No |
| 7 | NKTR |  |  | 1552 | -0.309 | -0.3309 | No |
| 8 | ZNF708 |  |  | 1702 | -0.318 | -0.3586 | No |
| 9 | PRH1 |  |  | 1783 | -0.324 | -0.3684 | No |
| 10 | MIR99AHG |  |  | 1829 | -0.326 | -0.3690 | No |
| 11 | ANKRD11 |  |  | 1878 | -0.328 | -0.3704 | No |
| 12 | DDX17 |  |  | 1950 | -0.332 | -0.3775 | No |
| 13 | LAMP2 |  |  | 1956 | -0.333 | -0.3676 | No |
| 14 | PCSK7 |  |  | 1959 | -0.333 | -0.3569 | No |
| 15 | CANX |  |  | 2209 | -0.349 | -0.4094 | No |
| 16 | ZNF431 |  |  | 2369 | -0.363 | -0.4382 | No |
| 17 | PDIA6 |  |  | 2505 | -0.373 | -0.4605 | No |
| 18 | ATP6AP2 |  |  | 2591 | -0.381 | -0.4696 | No |
| 19 | HMGCR |  |  | 2673 | -0.388 | -0.4775 | No |
| 20 | LMAN1 |  |  | 2743 | -0.394 | -0.4820 | No |
| 21 | FTX |  |  | 2927 | -0.412 | -0.5154 | No |
| 22 | GPM6A |  |  | 3171 | -0.440 | -0.5633 | No |
| 23 | HSPA5 |  |  | 3193 | -0.442 | -0.5539 | No |
| 24 | WSB1 |  |  | 3218 | -0.445 | -0.5451 | No |
| 25 | MSMO1 |  |  | 3241 | -0.450 | -0.5357 | No |
| 26 | CD63 |  |  | 3258 | -0.452 | -0.5246 | No |
| 27 | SLC1A3 |  |  | 3516 | -0.513 | -0.5737 | Yes |
| 28 | ADGRV1 |  |  | 3529 | -0.520 | -0.5593 | Yes |
| 29 | CALU |  |  | 3566 | -0.533 | -0.5507 | Yes |
| 30 | VMP1 |  |  | 3570 | -0.536 | -0.5335 | Yes |
| 31 | PLAT |  |  | 3600 | -0.547 | -0.5226 | Yes |
| 32 | SQLE |  |  | 3606 | -0.553 | -0.5053 | Yes |
| 33 | CD9 |  |  | 3634 | -0.567 | -0.4932 | Yes |
| 34 | FGFBP3 |  |  | 3649 | -0.578 | -0.4774 | Yes |
| 35 | COL4A2 |  |  | 3664 | -0.586 | -0.4613 | Yes |
| 36 | SOX6 |  |  | 3675 | -0.596 | -0.4439 | Yes |
| 37 | APLP2 |  |  | 3693 | -0.610 | -0.4278 | Yes |
| 38 | GRIA2 |  |  | 3721 | -0.633 | -0.4135 | Yes |
| 39 | HSP90B1 |  |  | 3757 | -0.681 | -0.3997 | Yes |
| 40 | APP |  |  | 3779 | -0.710 | -0.3812 | Yes |
| 41 | NCAM1 |  |  | 3821 | -0.773 | -0.3658 | Yes |
| 42 | COL4A1 |  |  | 3829 | -0.791 | -0.3411 | Yes |
| 43 | MIAT |  |  | 3833 | -0.794 | -0.3152 | Yes |
| 44 | KCNQ1OT1 |  |  | 3866 | -0.885 | -0.2937 | Yes |
| 45 | SCRG1 |  |  | 3869 | -0.904 | -0.2639 | Yes |
| 46 | TNC |  |  | 3874 | -0.951 | -0.2330 | Yes |
| 47 | NEAT1 |  |  | 3880 | -1.005 | -0.2005 | Yes |
| 48 | PTN |  |  | 3884 | -1.022 | -0.1670 | Yes |
| 49 | GPM6B |  |  | 3889 | -1.047 | -0.1328 | Yes |
| 50 | SNTG1 |  |  | 3909 | -1.316 | -0.0936 | Yes |
| 51 | PTPRZ1 |  |  | 3913 | -1.342 | -0.0493 | Yes |
| 52 | MALAT1 |  |  | 3924 | -1.583 | 0.0013 | Yes |
Table: GSEA details [plain text format]

  

Fig 2: LARSSON\_STATE\_2: Random ES distribution      
 Gene set null distribution of ES for **LARSSON\_STATE\_2**
